# Current News

**Published:** 1892-03

**Authors:** 


### Current News.

### MICHIGAN STATE BOARD OF EXAMINERS.

The annual meeting of the State Board of Examiners in Dentistry convened in the Senate chamber this afternoon for the purpose of the examination of the applicants for a license to practise dentistry in this State. The last Legislature made the College of Dental Surgery of the University of Michigan the standard of comparison in the examinations of this board. No diplomas are recognized from any institution whose requirements for graduation are not up to that of the university, and a resolution to that effect was adopted with reference to the applications of parties graduating from colleges whose requirements were below the standard at the present meeting. The fee for examination is ten dollars, and for registration without examination, for those holding diplomas from recognized colleges, three dollars.

The board consists of three members, all officers, and the doctrine of evolution is enforced by law in the organization. One member is appointed each year for the term of three years, and he, by law, becomes secretary of the board; the second year he becomes the treasurer, and the last year the president, and should he be reappointed he takes his place at the foot again. The present organization of the board is, President, A. T. Metcalf, Kalamazoo; Treasurer, H. K. Lathrop, Jr., Detroit; Secretary, G. E. Corbin, St. Johns.—*Detroit Free Press.*

Lansing, October 28.

---

### ST. LOUIS DENTAL SOCIETY.

The St. Louis Dental Society met at the residence of Dr. Conrad, on January 5, 1892, and elected the following officers for the ensuing year:

President, Dr. George Robitoy; Vice-President, Dr. Walter M. Bartlett; Recording Secretary, Dr. J. B. Vernon; Corresponding Secretary, Dr. John G. Harper; Treasurer, Dr. Henry Fisher.

*Committee on Publication.*—Drs. Helmuth, Lindsley, and Keith.

*Committee on Membership.*—Drs. Morrison, Hickman, and Spaulding.

*Committee on Ethics.*—Drs. Baird, Prosser, and McNamara.

The Executive Committee consists of the officers of the Society.

Regular meetings are held on the first Tuesday of each month, excepting July, August, and September.

John G. Harper,
*Corresponding Secretary.*

800 Pine Street, St. Louis, Mo.

---

### MISSISSIPPI VALLEY ASSOCIATION OF DENTAL SURGEONS.

The annual meeting of the Mississippi Valley Association of Dental Surgeons will take place March 8–11, 1892.

L. E. Custer,
*Secretary.*

---

### THE POST-GRADUATE DENTAL ASSOCIATION OF THE UNITED STATES.

The Post-Graduate Dental Association of the United States will hold its Annual Meeting, April 29 and 30 next, at the Leland Hotel, Chicago.

Dr. W. C. Barrett, of Buffalo, N. Y., Drs. T. W. Brophy, Louis Ottofy, and others, of Chicago, will present essays and addresses. An interesting programme has been arranged and a good attendance is expected. All members of the profession are invited.

Graduates of recognized Dental Colleges may become members by paying membership fee, one dollar, and dues for one year in advance, one dollar.

F. S. Tenney,
*Secretary.*

96 State Street, Chicago.

---

### ORAL SURGERY IN NEW YORK.

Dr. G. L. Curtis, of Syracuse, N.Y., announces in another place that he has established himself in New York as a specialist in oral surgery. The importance of this labor in our large cities can scarcely be over-estimated.

Dr. Curtis has laboriously prepared himself both at home and abroad in surgery, and doubtless will find in that city an ample field for the practice of his special work.

